# Allylation and Thermosetting of Acetosolv Wheat Straw Lignin

**DOI:** 10.1002/cssc.202402051

**Published:** 2024-12-17

**Authors:** Alessio Truncali, Davide Di Francesco, Cristiana Margarita, Iuliana Ribca, Louise Brandt, Benedikt Sochor, Stephan V. Roth, Mats Johansson, Helena Lundberg

**Affiliations:** ^1^ Department of Fibre and Polymer Technology KTH Royal Institute of Technology SE-100 44 Stockholm Sweden; ^2^ Wallenberg Wood Science Center (WWSC) Sweden; ^3^ Department of Chemistry KTH Royal Institute of Technology SE-100 44 Stockholm Sweden; ^4^ Deutsches-Elektronen Synchrotron (DESY) Notkestraße 85 22607 Hamburg Germany; ^5^ Advanced Light Source Lawrence Berkeley National Laboratory CA 94720 United States

**Keywords:** Wheat straw, Lignin, Thermoset, Allylation, Acetosolv

## Abstract

The acetosolv extraction, allylation and subsequent cross‐linking of wheat straw lignin to thermoset biomaterials is herein described. The extraction temperature proved to be of great importance for the quality of the resulting lignin, with moderate temperature being key for preservation of β‐O‐4’ linkages. The allylation of the acetosolv lignin was carried out using three different synthetic strategies, resulting in selective installation of either benzylic or phenolic allyl ethers, or unselective allylation of various hydroxyl groups via etherification and carboxyallylation. The different allylation protocols employed either allyl alcohol, allyl chloride, or diallylcarbonate as allyl precursors, with the latter resulting in the highest degree of functionalization. Selected allylated acetosolv lignins were cross‐linked using a thiol‐ene approach and the lignin with the highest density of allyl groups was found to form a cross‐linked thermoset material with properties comparable to kraft lignin‐based analogues.

## Graphical abstract

Acetosolv extraction of wheat straw lignin and subsequent functionalization towards thermoset polymers is described. The effect of extraction process and functionalization routes on the structure of the retrieved lignin is outlined, along with assessment of chemical properties and morphology of formed thermoset materials.

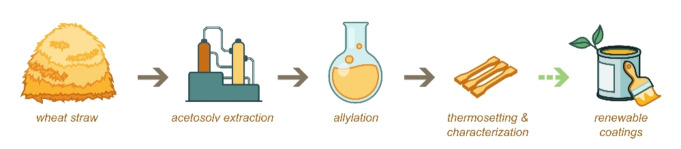

## Introduction

**Figure 1:**
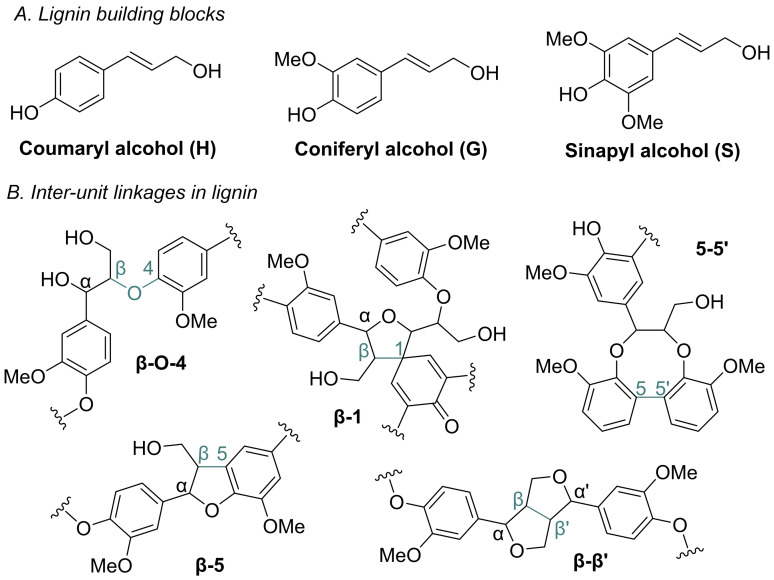
Structural features of lignin. A: Monolignols B: Common inter‐unit linkages.

Transformation of lignocellulosic biomass into functional compounds and materials is crucial to reduce the dependence of fossil feedstocks for consumer products. Methods for processing the carbohydrate fraction, for example, into pulp and paper as well as bioethanol, are well established. In contrast, lignin is currently mainly utilized as fuel in the same industry,^[1]^ although new applications have been emerging in recent years as the result of intense research. Such new applications of lignin include fillers,^[2]^ adhesives,^[3]^ packaging^[4]^ and biomedical materials.^[5]^ Furthermore, the great abundance of aromatic units makes the material an interesting candidate for thermostable bioplastics, including thermosets,^[6]^ as well as feedstock for organic compounds of low molecular weight via depolymerization procedures.^[7]^ Technical lignins, *e. g*. kraft lignin, soda lignin, and lignosulfonate, are side‐products in the pulp and paper industry and have been used as precursors for such cross‐linked materials.^[8]^ Lignin′s complex structure arises from its formation via radical coupling of the three primary monolignols: p‐coumaryl alcohol (p‐hydroxyphenyl‐H), coniferyl alcohol (guaiacyl‐G), and sinapyl alcohol (syringyl‐S) units.^[16]^ The composition of these units varies among plant and species, with softwood primarily consisting of G units with low levels of H units, hardwood containing both G and S units with traces of H units, and grass lignin consisting of a mix of G and S units with a higher proportion of H units (Figure 1A).[^16^, ^17^] Furthermore, the abundance of inter‐unit linkages, such as β‐O‐4, β‐5, β‐β′, and β‐1, 5–5′, varies with the source of the lignin (Figure 1B).

**Figure 2:**
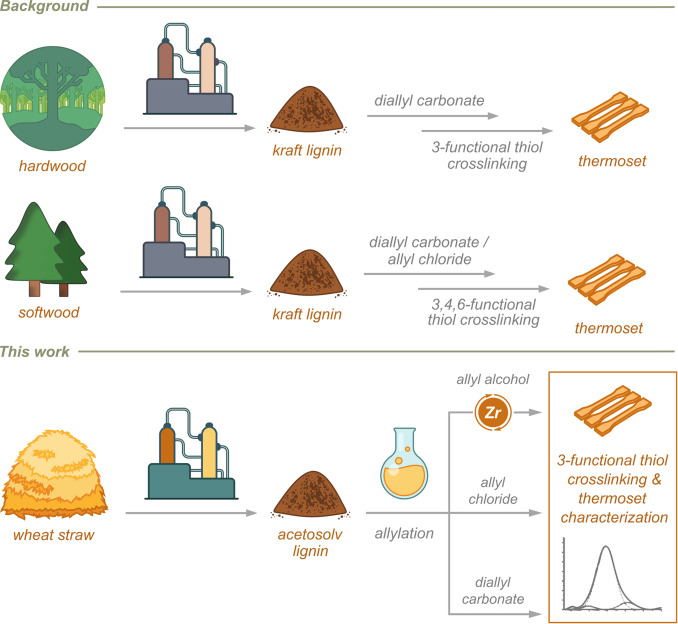
Routes to lignin‐based thermosets.

In addition to the compositional differences that result from the material′s biological origin, the chemical nature of the lignin is affected by the technique used to extract it from the parent lignocellulosic biomass. Commonly, such extraction is carried out via kraft, sulphite and soda pulping processes in the paper industry, producing technical lignins as side‐streams^[18]^ that are either used as fuel within the same industries[^1^, ^19^] or, to a lesser extent, used as starting materials for chemical valorization processes.[^3^, ^20^, ^21^, ^22^] These industrial pulping extraction procedures require harsh conditions that lead to degradation of the native lignin structure via, *e. g*., cleavage of the inter‐unit linkages. Innovative extraction techniques focus on preserving the natural lignin structure by achieving high lignin yield, processing at low temperatures, and minimizing structural alterations. These approaches aim to preserve the structural integrity of lignin, making it more suitable for high‐value applications. Solvent‐based extraction methods, such as those utilizing organic solvents under mild conditions, have shown promise in selectively extracting lignin while maintaining its structural integrity.[^23^, ^24^] Additionally, enzymatic treatments have emerged as a green and effective approach, offering the advantage of high specificity in breaking down lignin‐carbohydrate complexes without extensive chemical modification of lignin.^[25]^ Deep eutectic solvents (DES) and ionic liquids (IL) are also being investigated for their ability to dissolve lignin under milder conditions, thus offering an alternative to traditional pulping processes.^[26]^ To date, the majority of lignin‐based products rely on streams of wooden origin. In contrast, the use of lignocellulose from annual plants is considerably less explored in a biorefinery setting, even though agricultural side‐streams such as straw, husks, peels and stovers are produced in most countries worldwide. As such, the importance of crop residues is expected to increase as renewable feedstocks for the production of functional compounds and materials in the coming decades. Wheat straw is annually generated in abundance worldwide (~500 million tons/year) and methods for its fractionation into its principal constituents have been developed using, *e. g*., steam explosion, wet oxidation, acid hydrolysis, organosolv and alkali treatment as well as microwave‐assisted procedures and the use of alternative solvents.[^25^, ^27^, ^28^, ^29^] Nevertheless, wheat straw lignin is still largely unutilised due to the lack of an industrially relevant extraction method.^[30]^ In the context of coatings, lignin can form durable, adhesive and resistant protective materials with applications such as UV curing agents,^[31]^ polymer nanocomposites,^[32]^ anticorrosive agents,[^33^, ^34^] antimicrobial agents,^[35]^ antifoulant agents,^[36]^ water repellents^[37]^ and flame retardants.^[38]^ To enable such use, chemical modification of the lignin is generally required. One such modification is the installation of allyl ether moieties that can be used as functional handle for subsequent cross‐linking via thiol‐ene click chemistry.[^8^, ^13^] In this context, different strategies using a variety of allylating reagents have been developed. These reagents exhibit different selectivity towards functional groups in the lignin and, hence, result in different degrees of allylation of the material. One of the most effective reported electrophilic allylating agents is diallyl carbonate, reported to react with both aromatic and aliphatic hydroxyl groups in technical lignins.[^39^, ^40^, ^41^, ^42^, ^43^] Furthermore, allyl halides have been used as electrophilic allylating agent under basic conditions, furnishing highly selective allylation of phenols over aliphatic alcohols in kraft lignin.[^8^, ^13^, ^34^, ^35^, ^36^, ^37^, ^38^] In addition, this strategy was used for allylation of industrial lignin obtained from agricultural fibrous feedstocks.^[44]^ The technical lignins allylated using these methods were subsequently cross‐linked to thermosets using thiol‐ene chemistry (Figure 2). In addition, we recently disclosed a Lewis‐catalyzed etherification procedure that utilizes allyl alcohol as a nucleophilic allylating agent, resulting in selective functionalization of electrophilic benzylic sites in wheat straw lignin.^[42]^ In this work, we extracted lignin from wheat straw by means of an acetosolv procedure, allylated the lignin using these three different procedures, subjected the resulting materials to cross‐linking conditions via thiol‐ene click chemistry and assessed the properties of the resulting materials (Figure 2). The degree of allylation was found to correlate closely with the choice of allylation method, as was the ability of the lignin material to form crosslinked thermosets. Furthermore, comprehensive characterization via spectroscopic, thermomechanical and X‐ray based techniques elucidated the correlations between nanoscale and macroscale properties of the formed wheat straw lignin thermoset.

## Results and Discussion

### Lignin Extraction

**Figure 3:**
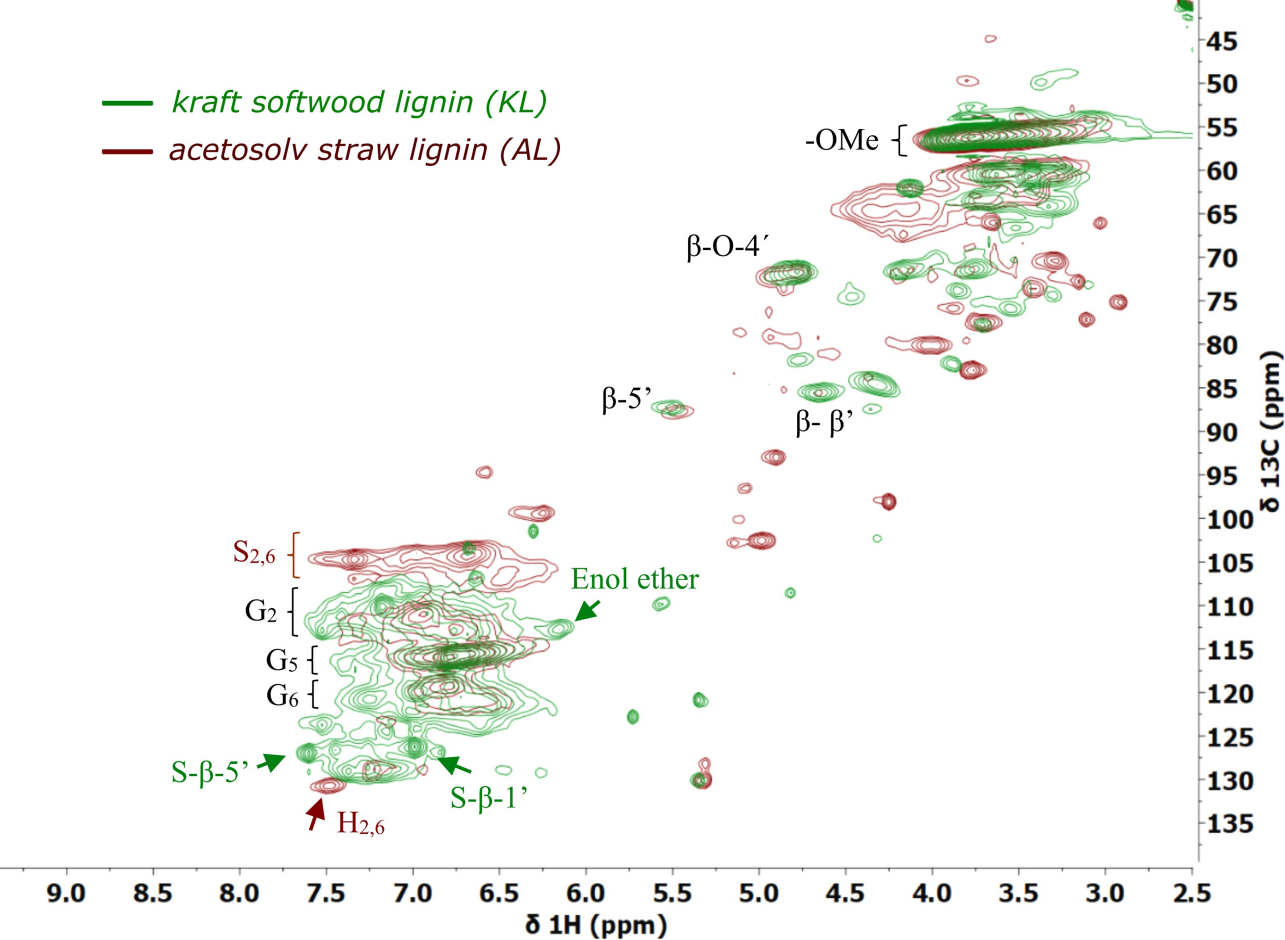
2D HSQC overlapped spectra of acetosolv straw lignin (AL, red) and Kraft softwood lignin (KL, green). The red abbreviations are structures attributed only to AL, the green texts are structures attributed only to KL, the black abbreviations are shared structures present in both lignins. See Figure 1 for details on structural elements.

Among the biomass fractionation technologies available for wheat straw, an acetosolv extraction was chosen due to its proven scalability and the relatively low degradation of the corresponding lignin along with a high benzylic alcohol content.[^45^, ^46^] For the extraction, the cut straw was refluxed in a mixture of formic acid, acetic acid and water, followed by removal of the pulp by filtration. The acidic liquor was thereafter treated with water to precipitate the lignin, with or without prior removal of organic acids by distillation under reduced pressure. The precipitated lignin was filtered off, washed with water, centrifuged and finally freeze‐dried to furnish a clean substrate for the study (see ESI, section 2.1 for details). Furthermore, the acetosolv procedure could easily be scaled from two grams of wheat straw to 120 grams to furnish AL of comparable quality (see ESI section 2.1 for details). The acetosolv lignin (AL) was analysed by HSQC nuclear magnetic resonance (NMR) spectroscopy, demonstrating that the material was rich in guaiacyl (G) and syringyl (S) units with a S/G ratio of 0.5 along with a small amount of *p*‐hydroxyphenyl (H) (see ESI, Figures S1–4 and Tables S1–2). Furthermore, the material was found to contain interunit linkages that consisted of β‐O‐4 (α‐OH and α‐keto) linkages to around 67 %. Due to the relative lability of this linkage, its relatively high prevalence is a testimony of the mildness of the extraction procedure as compared to kraft‐type conditions.^[3]^ Similarly, the relatively balanced distribution of hydroxyl groups in AL as determined by ^31^P‐NMR (2.49 mmol phenolic OH/g_lignin_, 1.27 mmol aliphatic OH/g_lignin_ and 0.51 mmol carboxylic OH/g_lignin_) (Figure S8) suggests that extensive phenolic ether bond cleavage is avoided with the applied extraction conditions, in contrast to what is observed for kraft lignin that displays a significantly higher phenol content.^[41]^ Compared to commercially available kraft softwood lignin (KL) – arguably the benchmark material for lignin studies – HSQC analysis displays distinct structural differences (Figure 3). Specifically, the AL sample showed significant signals corresponding to syringyl (S_2,6_) and p‐hydroxyphenyl (H_2,6_) units, while the KL sample almost exclusively exhibited guaiacyl units (G_2_, G_5_, G_6_) (~100 %). Furthermore, the presence of stilbene elements (S‐β‐5′ and S‐β‐1′) was detected in KL, in contrast to AL. The occurrence of these side‐chains as well as enol ethers, β‐5′, and β‐β′ linkages reduces the relative amount of β‐O‐4′ linkages compared to the relative prevalence of this moiety in AL. Significant differences between KL and AL were also found in the carbohydrate region.^[47]^ These results correlate well with previous studies of straw lignin.[^17^, ^48^] Further characterization details of AL and comparisons with KL are found in ESI (Secti).

### Lignin Allylation

**Figure 4:**
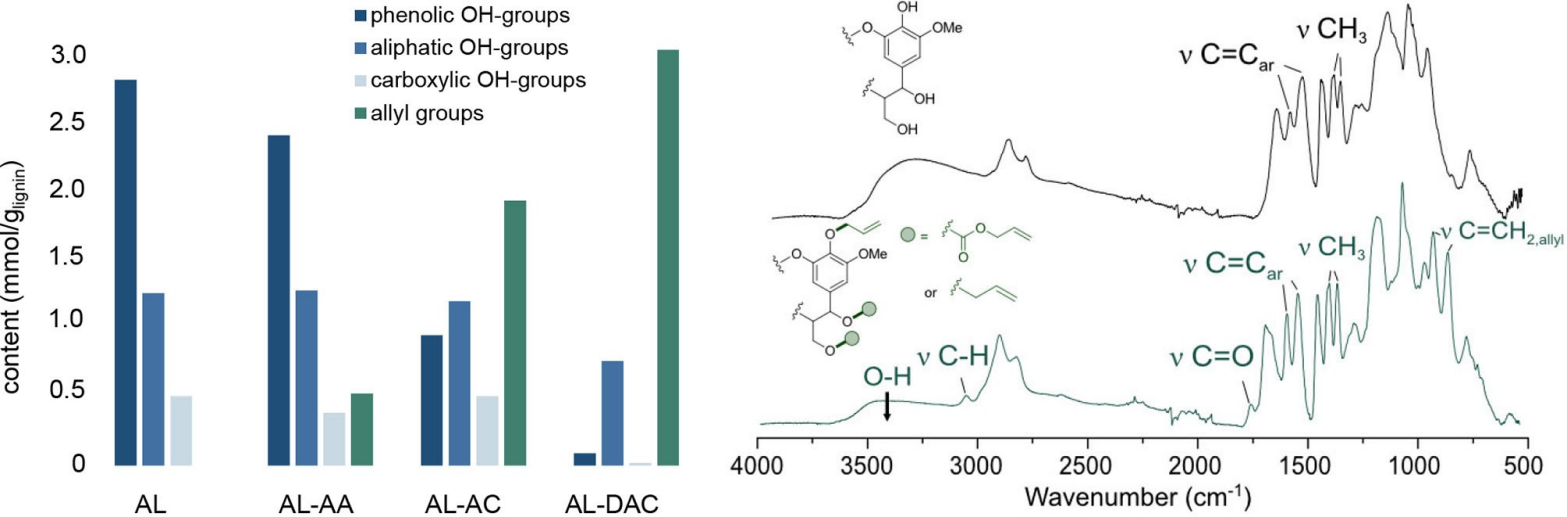
Chemical characterization of unfunctionalized (AL), allyl alcohol functionalized (AL‐AA), allyl chloride functionalized (AL‐AC), and diallyl carbonate functionalized (AL‐DAC) acetosolv lignin samples. Left: Quantification of OH‐ and allyl groups using ^31^ P‐NMR and ^1^H‐NMR, respectively (see ESI, Figures S8–9, Tables S5–6 for details). Right: FTIR spectra of unmodified lignin (AL) on top and diallyl carbonate functionalized lignin (AL‐DAC) on the bottom.

To furnish lignin materials with different degrees of allylation, AL was subjected to three different allylation procedures (Figure 4). Procedure 1 used our previously developed Zr‐catalysed protocol with selectivity towards allylation of benzylic sites, using allyl alcohol (AA) as reagent to furnish the allylated lignin AL‐AA (see ESI, section 2.2 for details).^[42]^ In procedure 2, allylated lignin was synthesized following our previously reported base‐mediated procedure with allyl chloride (AC) as allylating agent,^[8]^ furnishing allylated lignin AL‐AC (see ESI, section 2.3 for details). This method was chosen due to its selectivity for allylation of phenolic sites. Finally, procedure 3 used diallyl carbonate (DAC) as allylating agent with ability to functionalize both aromatic and aliphatic OH‐groups under phase transfer conditions to furnish the allylated material AL‐DAC (see ESI, section 2.4 for details).[^39^, ^40^, ^41^] The allylated AL materials were analyzed using ^31^P NMR spectra and compared to unmodified AL to elucidate the chemoselectivity of the different allylation methods. As evident from Figure 4, AL allylated with allyl alcohol (AL‐AA) contained a modest concentration of allyl groups (~0.5 mmol/g) whereas AL functionalised with allyl chloride (AL‐AC) displayed a significantly higher concentration of allyl groups (~1.9 mmol/g). In both cases, the increase in allyl content was correlated to the decrease in aromatic OH‐groups.^1^ Finally, AL allylated using diallyl carbonate (AL‐DAC) exhibited the highest allyl content (~3.1 mmol/g) and the lowest total remaining OH‐content, reflecting that an effective functionalization of various types of hydroxyl groups in the material had occurred (Figure 4, left). The structural features in the non‐allylated AL and AL‐DAC was further studied using Fourier‐transfer infrared (FTIR) spectroscopy (Figure 4, right). As expected, AL displayed a prominent peak associated with O‐H bond stretching vibrations in the range between 3600 and 3200 cm^−1^, whereas the intensity of this peak was significantly reduced in AL‐DAC. At the same time, an increase in intensity in the signals for C‐H bonds was observed for AL‐DAC compared to AL. Notably, a new peak emerged at 3079 cm^−1^ in AL‐DAC that can be attributed to C‐H bond stretching vibrations of the allyl group. Furthermore, analysis of the carbonyl region revealed notable changes in the allylated AL‐DAC compared to AL with a new peak at 1770 cm^−1^ in the former material, indicative of carboxyallylation which occurs at aliphatic OH‐groups.[^38^, ^39^, ^40^] Finally, the presence of new peaks at 987 cm^−1^ and 922 cm^−1^, attributed to C‐C bending vibrations, in AL‐DAC are further confirming the presence of allyl groups in the functionalized lignin.^[39]^ Details and further characterization data can be found in ESI (Figures S7‐S9 and Tables S5‐S7).

### Thermoset Formation

**Figure 5:**
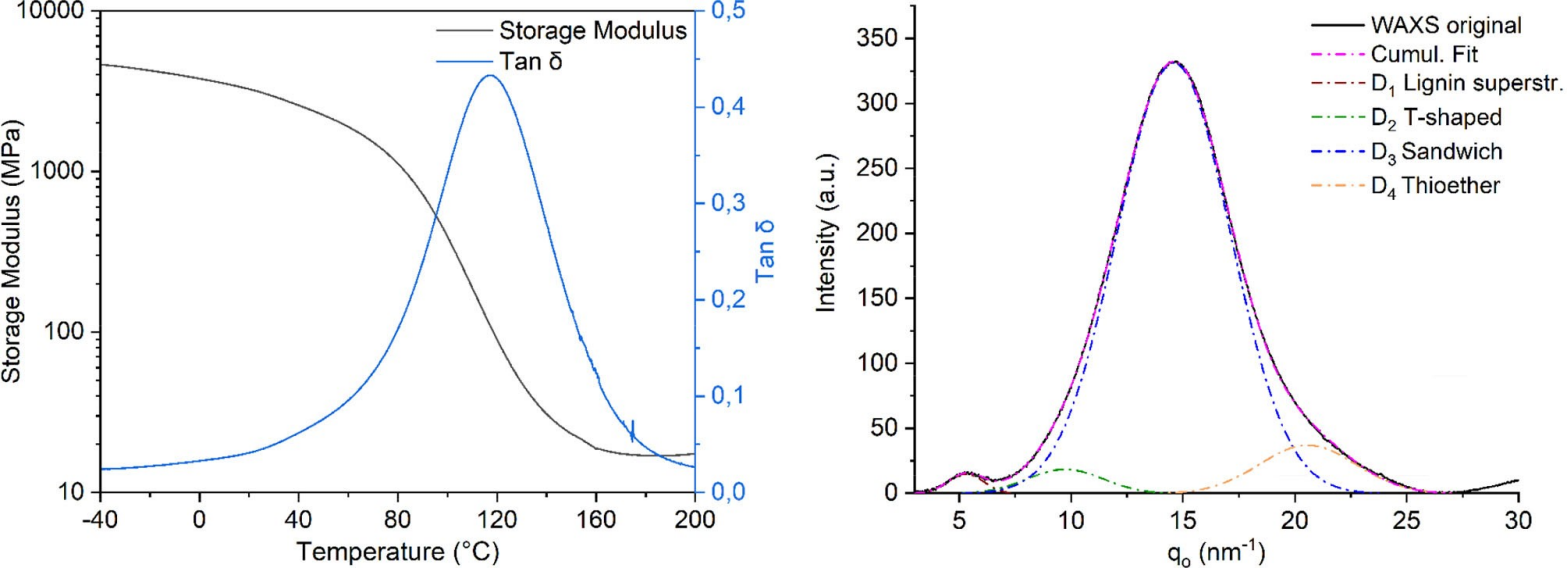
Properties of T‐AL‐DAC. Left: Dynamic mechanical analysis (DMA) showing the storage modulus (E′) and tan delta (δ) as functions of temperature (°C). Right: Wide‐angle X‐ray scattering (WAXS) analysis indicating secondary interactions within the thermoset.

For thermosetting applications via thiol‐ene click chemistry, high allyl content in the uncured resin is generally beneficial. Indeed, when the allylated lignin with the lowest degree of functionalization – AL‐AA – was subjected to cross‐linking conditions, it was unable to form a thermoset material. In contrast, AL‐AC that displayed a higher degree of allylation could be converted to a thermoset, but this material was found too brittle for mechanical properties to be assessed. However, AL‐DAC that contained the highest allyl content was found to smoothly form a rigid thermoset material (T‐AL‐DAC). FTIR before and after curing shows that thiol groups were fully converted (see ESI, Figure S12). This material was assessed using dynamic mechanical analysis (DMA) to investigate its thermo‐mechanical properties and viscoelastic behavior (Figure 5, left). At −40 °C, a relatively high storage modulus (E′) of around 4.7 GPa was found, in line with properties previously determined for other thermosetting lignin systems.[^13^, ^37^] A storage modulus above 4 GPa is suitable for several applications, such as composite matrices and organic coatings where a high stiffness is required. Furthermore, T‐AL‐DAC displayed a glass transition temperature (T_g_) at 117±5 °C, calculated from the peak of tan δ. This value is in the same range as that of thermosets based on technical hardwood lignin^[41]^ and higher than thermosets based on technical softwood lignin by around 20–30 °C.^[51]^ This behavior may be understood in light of structural features of the thermoset network. Similar to hardwood and in contrast to softwood analogues, the wheat straw lignin contains a relatively high content of syringyl units (ESI, Table S1). These syringyl units contribute to a relatively high degree of methoxylation of the material and likely contributes to a more rigid structure due to locked chain conformations due to steric hindrance. In turn, this increased stiffness results in a higher glass transition temperature. Furthermore, the technical softwood lignin obtained via a kraft‐type pulping procedure contains a higher degree of free hydroxyl groups compared to our AL. Thus, when the OH‐rich softwood lignin is allylated using DAC[^39^, ^41^] and subsequently thermoset via formation of flexible aliphatic thioether linkages, the resulting thermoset can be expected to display a more supple nature and a lower T_g_ compared to T‐AL‐ DAC. Furthermore, the T_g_ of a thermoset will be affected by factors such as crosslink density, flexibility of the crosslink, and the nature of secondary interactions. The crosslinking with thioether linkages introduces flexible crosslinks that may reduce the T_g_ by its presence but this is to some extent compensated by the increased crosslink density. The interplay between crosslink density, thioether linkages in combination with the presence of the ordered aromatic structures in the lignin precursor result in a complex system, with the π‐π stacking interactions between aromatic units being known to significantly impact  morphologies and physical properties of lignin materials.[^8^, ^13^, ^41^, ^51^] To understand the morphology of T‐AL‐DAC, wide‐angle X‐ray scattering (WAXS) analysis was carried out (Figure 5, right and Table S8 in ESI) and the the extracted WAXS curves were fitted with four Gaussians functions. The Gaussian peaks were found at distances D_1_=11.88±0.21 Å, D_2_=6.45±0.15 Å, D_3_=4.3±0.005 Å and D_4_=3.05±0.02 Å, calculated from reciprocal space (nm^−1^) according to formula reported in ESI (section 3.2). D_2_ and D_3_ could be attributed to T‐shaped and sandwich π‐π interactions, respectively (Figure 3, right), in analogy to what has previously been reported.[^12^, ^13^, ^41^, ^48^, ^49^] The ratio of sandwich to T‐shaped π‐π stacking interactions was determined to be ~26±4:1. Since sandwich interactions are known to contribute to increased stiffness, whereas T‐shaped interactions are generally associated with higher flexibility,^[50]^ this result may partially explain the relatively high glass transition temperature and rigidity of T‐AL‐DAC. The D_1_, at longer distance, can be related to intra‐ or intermolecular superstructures that can be found in native lignin.[^51^, ^52^] A new signal close to the D_4_ distance of the present study was also found in lignin‐based thermosets of a previous study.^[51]^ Due to the high amount of thiol monomer in the formulation, this signal is proposed to be attributed to a phase rich in thioether moieties from the crosslinking. In summary, the overall macroscopic mechanical properties are determined by a number of different structural features that all contribute in different ways on different length scales. The sandwich π‐π stacking provides rigidity and strength in the short distance range while the T‐shaped interactions provide more flexibility, although still representing a rigid phase. The longer distances related to superstructures are less pronounced and are proposed to correlate to the domains of the rigid lignin clusters. In‐depth understanding on how these clusters affect the macroscopic performance remain the subject of further studies. The short distance range related to the thioether‐rich phase is however more pronounced, likely providing more flexibility to the material due to the intrinsic properties of the thioether linkage. Details on synthetic procedures and characterization of the thermosets are found in ESI (Section 3).

## Conclusions

To the best of our knowledge, this work presents the first example of a lignin‐based thermoset material derived from wheat straw, showcasing how agricultural residue can be chemically converted into functional materials. Specifically, we describe mild lignin extraction using a scalable acetosolv procedure, an efficient allylation procedure using the environmentally friendly reagent diallyl carbonate and finally a curing procedure via thiol‐ene click chemistry. The resulting thermoset material was found to display a relatively high glass transition temperature and stiffness, similar to that previously observed for kraft hardwood analogues, making it fit for potential applications in, *e. g*., composite matrices and organic coatings. Morphological studies using wide‐angle X‐ray scattering (WAXS) correlate the relatively high glass transition temperature and rigidity to a high degree of π‐π stacking sandwich interactions in the wheat straw‐derived thermoset material.

## Author Contributions

A.T.: Conceptualization, Data curation, Formal analysis, Investigation, Visualization, Writing – Original Draft Preparation, Writing – Review & Editing.

D. D. F.: Conceptualization, Data curation, Formal analysis, Investigation, Methodology, Validation, Visualization, Writing – Review & Editing.

C. M.: Data curation, Project administration, Visualization, Writing – Review & Editing.

I. R.: Data curation, Formal analysis, Investigation, Writing – Review & Editing.

L. B.: Data curation, Formal analysis, Investigation, Writing – Review & Editing.

B. S.: Formal analysis, Investigation, Writing – Review & Editing.

S. V. R.: Formal analysis, Investigation, Writing – Review & Editing.

M. J.: Conceptualization, Data curation, Funding acquisition, Project administration, Supervision, Writing – Original Draft Preparation, Writing – Review & Editing.

H. L.: Conceptualization, Data curation, Funding acquisition, Project administration, Supervision, Writing – Original Draft Preparation, Writing – Review & Editing.

## Conflict of Interests

The authors declare no conflict of interest.

1

## Supporting information

As a service to our authors and readers, this journal provides supporting information supplied by the authors. Such materials are peer reviewed and may be re‐organized for online delivery, but are not copy‐edited or typeset. Technical support issues arising from supporting information (other than missing files) should be addressed to the authors.

Supporting Information

## Data Availability

The data that support the findings of this study are available in the supplementary material of this article.

## Footnotes

n1 This finding is in line with previous studies using allyl chloride as allylating agent, but stands in interesting contrast to previous work using allyl alcohol as allylating agent in which phenols were found to unreactive under the Lewis acid‐catalysed conditions. While the origin of this reactivity difference was not the subject of further study, the present results underscores the impact that structural differences in lignin can have.
