# Supplementary material for: Allylation and Thermosetting of Acetosolv Wheat Straw Lignin
Source: ChemSusChem. 2024 Dec 17;18(7):e202402051. doi: 10.1002/cssc.202402051 (PMC11960581; doi:10.1002/cssc.202402051)
Supplement: Supplementary file 1 — Supporting Information [file CSSC-18-e202402051-s001.pdf]

ChemSusChem

Supporting Information

Allylation and Thermosetting of Acetosolv Wheat Straw Lignin

Alessio Truncali, Davide Di Francesco, Cristiana Margarita, Iuliana Ribca, Louise Brandt, Benedikt Sochor, Stephan V. Roth, Mats Johansson,* and Helena Lundberg*

Supporting Information

Allylation and Thermosetting of Acetosolv Wheat Straw Lignin

Alessio Truncali,‡[a] Davide Di Francesco,‡[b] Cristiana Margarita,[b] Iuliana Ribca,[a] Louise Brandt,[a] Benedikt Sochor,[c], [d] Stephan V. Roth,[a], [c], Mats Johansson,[a]* Helena Lundberg[]*

[a] Department of Fibre and Polymer Technology
KTH Royal Institute of Technology
SE-100 44, Stockholm Sweden
E-mail: matskg@kth.se

[b] Department of Chemistry
KTH Royal Institute of Technology
SE-100 44, Stockholm, Sweden
E-mail: hellundb@kth.se

[c] Deutsches-Elektronen Synchrotron DESY
Notkestr. 85
22607 Hamburg, Germany

[d] Advanced Light Source
Lawrence Berkeley National Laboratory
CA 94720, United States

‡ These authors contributed equally to this work.

Contents

1. General information **2**

1.1 Materials 2

1.2 Methods 2

1.2.1. Phosphorus and Proton Nuclear Magnetic Resonance Spectroscopy (^{31}P NMR) and (^{1}H NMR). 2

1.2.2. Heteronuclear Single Quantum Coherence (HSQC) Spectroscopy 2

1.2.3. Fourier Transform Infrared Spectroscopy (FTIR) 3

1.2.4. Size Exclusion Chromatography (SEC) 3

1.2.5. Dynamic Mechanical Analysis (DMA) 3

1.2.6. Wide angle X-ray scattering (WAXS) 3

2. Synthetic procedures and characterization data **3**

2.1. General acetosolv procedure for extraction of wheat straw lignin 3

2.2. Allylation procedure 1 (Zr catalysis + allyl alcohol) 11

2.3. Allylation procedure 2 11

2.4 Allylation procedure 3 11

3. Thermosets **15**

3.1 Thermoset formulations and characterization 15

3.2. WAXS characterization 16

4. References **17**

Abbreviations

- AL = Acetosolv extracted straw lignin
- KL = Softwood kraft lignin
- AL-AA = Allylated lignin with allyl alcohol procedure
- AL-AC = Allylated lignin with allyl chloride procedure
- AL-DAC = Allylated lignin with diallyl carbonate procedure
- T-AL-DAC = Thermoset from allylated lignin with diallyl carbonate procedure

1. General information

1.1 Materials

Solvents and reagents were purchased from commercial suppliers and used without further purification. Technical softwood kraft lignin was provided by Stora Enso and used as received. Wheat straw was provided by Lantmännens forskningsstiftelse. Reactions were conducted under ambient atmosphere with magnetic stirring with heating in an oil bath equipped with temperature regulator unless otherwise noted.

1.2 Methods

1.2.1. Phosphorus and Proton Nuclear Magnetic Resonance Spectroscopy (^{31}P NMR) and (^{1}H NMR).
Spectroscopic analyses were carried out on lignin samples using a Bruker Avance III HD 400 MHz instrument with a Z-gradient coil. The spectra were phase and baseline, (Bernstein Polynomial Fit) corrected.
Quantitative analysis of hydroxyl groups in lignin was performed via ^{31}P NMR. Approximately 25 mg of lignin was dissolved in a pyridine/DMF and 1/10 of an internal standard solution (50 µl) prepared by dissolving the internal standard, N-hydroxy-5-norbornene-2,3-dicarboximide (NHND, 97%) (30 ± 0.1 mg), and (2.5 ± 0.2 mg) of a relaxation reagent (chromium(III) acetylacetonate) in anhydrous pyridine (500 µl). The samples were reacted with 2-chloro-4,4,5,5-tetramethyl-1,3-2-dioxaphospholane (100 µl).[53] The spectra were recorded 20 min after preparation using an inverse gated decoupling pulse (zgig 30) with a relaxation delay of 10 s, 128 number of scans, and an acquisition time of 4.9 s. The signal of the solvent, deuterated chloroform (400 µl), at 132.2 ppm was used as reference signal for the chemical shift. The signal at 151.8 ppm was attributed to the internal standard, with its integral normalized to a value of 1.
^{1}H NMR was performed in order to quantify allyl groups before and after allylation procedures dissolving 12.2 of 4-nitrobenzaldehyde as internal standard. Lignin samples (20 ± 0.1 mg) and the internal standard were dissolved in 550 µL DMSO-d6. The spectra were recorded with a relaxation delay of 5 s, a number of scans of 32, and an acquisition time of 4.9 s. The solvent signal at 2.50 ppm was used as a reference signal for the chemical shift. The signal at 10.16 ppm was assigned to the internal standard, with its integral normalized to 1. Two additional signals at 8.40 and 8.17 ppm were also attributed to the internal standard but were not used for quantification.

1.2.2. Heteronuclear Single Quantum Coherence (HSQC) Spectroscopy
A Bruker Avance III HD 400 MHz instrument was utilized to semi-quantify lignin inter-unit linkages. About 80 mg of lignin was dissolved in deuterated dimethyl sulfoxide. Spectra were collected using the pulse program “hsqcetgpsi” with an acquisition time of 0.0625, a relaxation delay of 1.5 s and 64 scans using a relation delay of 1.5 s. Signals have been assigned from previous studies.[8-16]

1.2.3. Fourier Transform Infrared Spectroscopy (FTIR)
Structural changes in lignin were examined with a PerkinElmer Spectrum 100 FTIR instrument in ATR mode, equipped with a diamond crystal at 16 scans. FTIR spectra were obtained at room temperature.

1.2.4. Size Exclusion Chromatography (SEC)
The molar mass and dispersity of lignin samples were determined using a SECurity 1260 Infinity GPC System, equipped with a refractive index detector and PSS GRAM precolumn and separation columns. The analysis was conducted at 60°C, using dimethyl sulfoxide with 0.5% lithium bromide as the eluent at a flow rate of 0.5 mL/min. Lignin samples were dissolved at a concentration of 5 mg/mL, filtered through a 0.45 µm syringe filter, and calibrated against pullulan standards.

1.2.5. Dynamic Mechanical Analysis (DMA)
DMA was carried out using a TA Instruments DMA Q800 device in tensile mode. The sample, with dimensions 6.5 × 6.9 × 1 mm, retained its geometry from the mold. The analysis began at a temperature of -40 °C, which was maintained isothermally for 10 minutes before heating at a rate of 3 °C/min up to 200 °C. The frequency used was 1 Hz, with a strain amplitude of 6.5 µm.

1.2.6. Wide angle X-ray scattering (WAXS)
Wide-angle X-ray scattering measurements were conducted at beamline P03 (PETRA III, Hamburg, Germany) [40]. The lignin thermosets (6.5 mm x 7.0 mm x 1 mm) were measured as-is. A LAMBDA 9 M (X-spectrum, Hamburg, Germany) detector with a fixed X-ray wavelength of 1.048 Å (E = 12.12 keV, λ = 1.023 Å) was utilized. The sample-to-detector distance (SDD) was 21 cm with a beam size of 35 × 28 μm^2. To prevent beam damage from the X-rays, the radiation dose was distributed by scanning 100 different positions over a 2 × 2 mm^2 region, with each position illuminated for 1 second. The acquired images were summed and reduced to 1D scattering profiles through azimuthal integration and normalization, as described in the literature, followed by background subtraction (air scattering). The scattering profiles display radially averaged intensities over the scattering vector $q = (4\pi/\lambda) \sin \theta$, where is the X-ray wavelength and θ is the scattering angle measured from the point where the primary beam hits the detector. These profiles were fitted using a combination of Gaussian functions. The obtained peak positions q* in reciprocal space were then recalculated to determine the D-spacing ($D = 2\pi/q^*$), which relates to the distances and sizes of repeating features in real space.

2. Synthetic procedures and characterization data

2.1. General acetosolv procedure for extraction of wheat straw lignin
The acetosolv lignin extraction was adapted from a reported procedure.[46] 120 g of oven dried wheat straw were put into a mixture containing 1650 mL of formic acid, 900 mL of acetic acid and 450 mL H_2O in a 5-liter round bottom flask fitted with an open condenser. Magnetic stirring was maintained and the suspension was refluxed at a temperature of 105°C for 3.5 hours. The mixture was cooled down to *ca.* 40° C and the acidic solution separated from the pulp by filtration and pressing. The pulp was then washed with hot water. The lignin and sugar liquors mixture were subjected to distillation under reduced pressure to remove as much formic acid as possible and then treated with H_2O (5 L). Alternatively, the addition of a larger volume of H_2O (20 L) was performed directly for lignin precipitation without distilling off the organic acids. The precipitated lignin was separated off by filtration, washed with H_2O (3x 30 mL), centrifuged and then freeze-dried to furnish 13 g of lignin (AL).

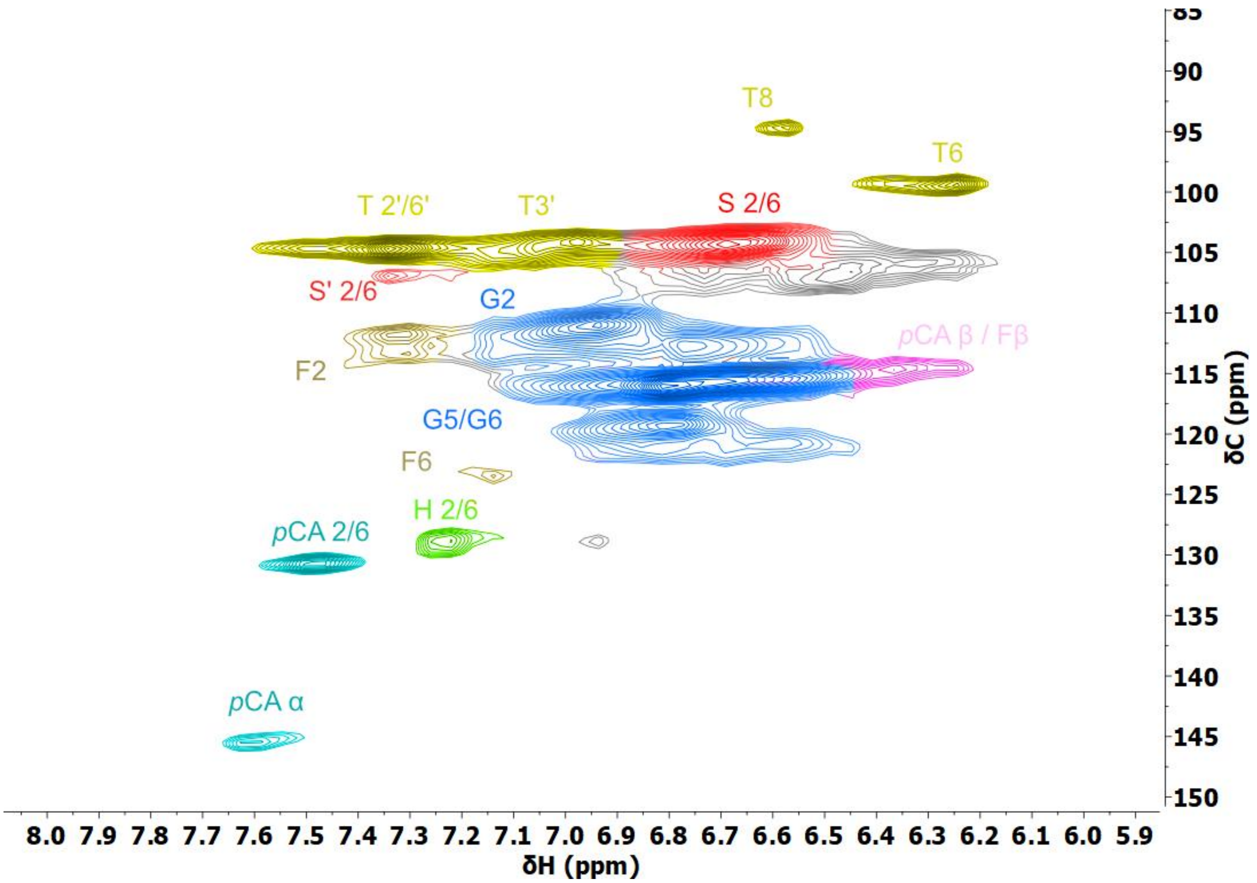

Figure S1. Aromatic/unsaturated (δC/δH 85−150/6.0−8.0) regions in the 2D HSQC NMR spectra of acetosolv wheat straw lignin (AL).

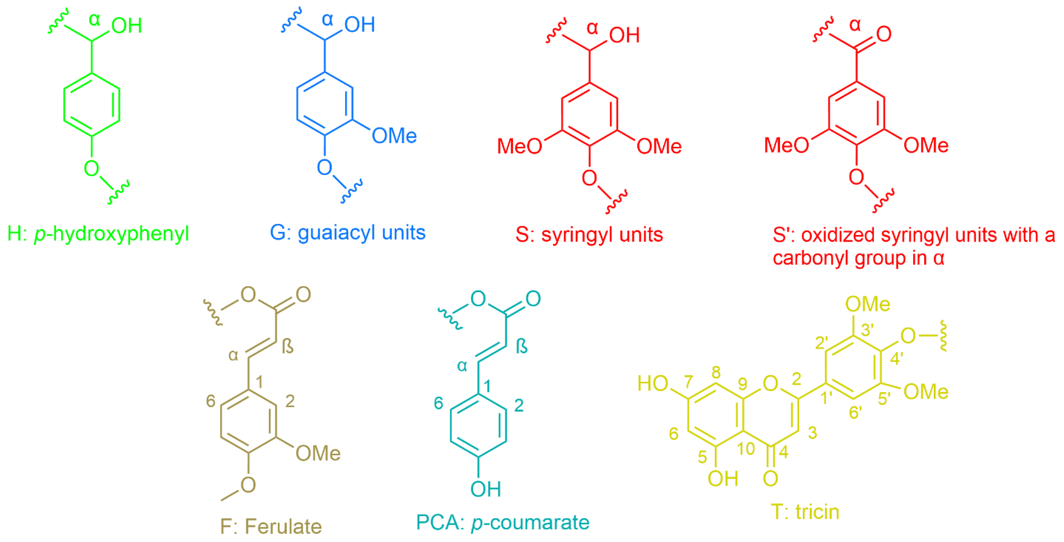

Figure S2. Main lignin aromatic/unsaturated structures identified in acetosolv wheat straw lignin (AL).

Table S1. Signal assignments of aromatic/unsaturated structures in AL.[17,43]

**Aromatic moiety**	**Abbreviation**	**δC**	**δH**	**%**
**Monolignols aromatic units[a,b]**				
Syringyl (S+S')	S2/6 (symmetric)	107.8-101.8	6.9-6.4	31.8
	S'2/6 (symmetric)	108.8-105.9	7.4-7.1	
Guaiacyl (G)	G2	114.2-108.2	6.9-6.4	64.0
	G5	117.1-114.3	7.1-6.4	
	G6	123.5-116-9	7.1-6.3	
p-Hydroxypheyl (H)	H2/6 (symmetric)	130.9-127.1	7.4-6.8	4.2
S/G ratio	0.5			
**Hydroxycinnamates (HC)[c]**				
p-Coumarate (*p*CA)	*p*CA2/6 (symmetric)	132.0-129.4	7.7-7.3	4.3
	*p*CAα	146.6-143.8	7.7-7.5	
	*p*CAβ overlapped Fβ	116.2-113.3	6.4-6.1	
Ferulate (F)	F2	114.0-110.2	7.4-7.2	6.1
	F6	125.3-121.3	7.3-7.0	
	Fβ overlapped *p*CAβ	116.2-113.3	6.4-6.1	
HC Total				10.4
**Lignan**				
Tricin (T)	T2'/6' (symmetric)	106.3-102.5	7.5-7.2	7.4
	T3	106.3-102.9	7.1-6.9	
	T6	101.0-97.7	6.4-6.1	
	T8	96.9-92.3	6.7-6.4	

[a] The percentage represent all aromatic monolignols (S+S'+G+H = 100%).
[b] All integrals were calculated by normalizing G2 integral to 100.
[c] Hydroxycinnamates and tricin have been expressed as percentage of all aromatic monolignols (S+S'+G+H).

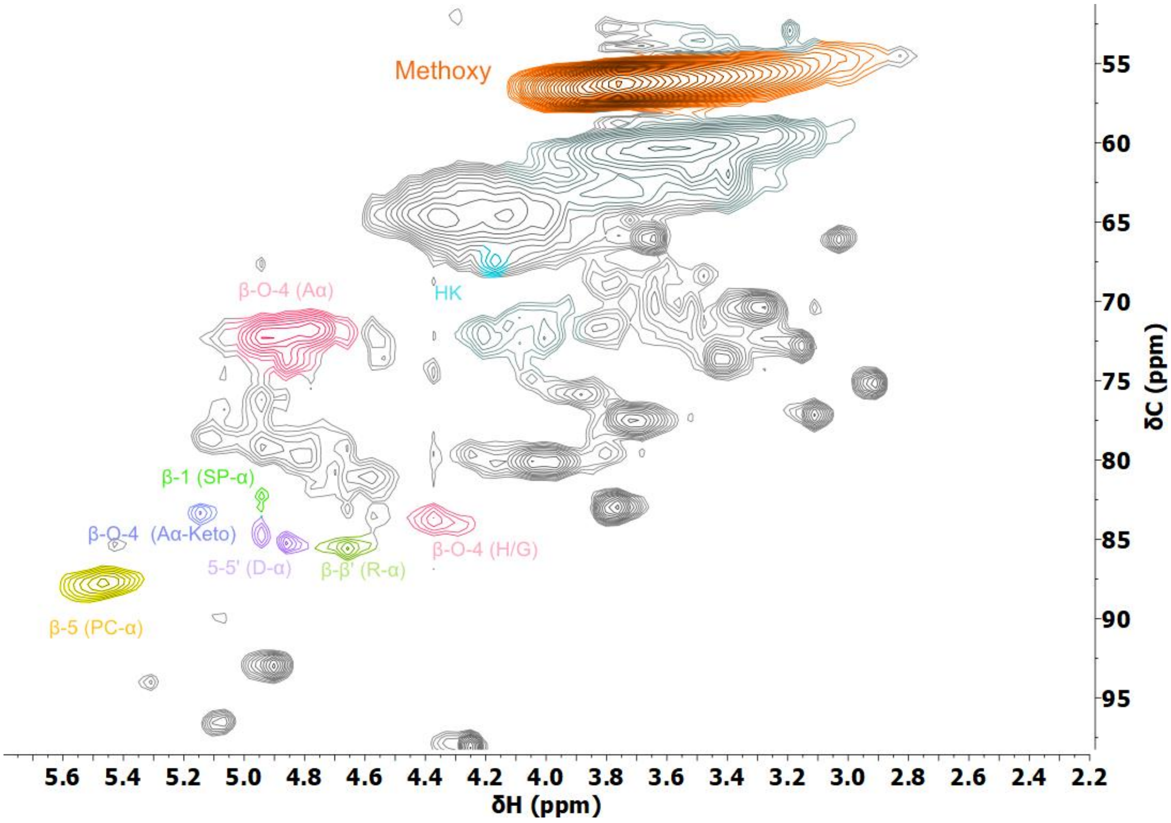

Figure S3. Side chain (and carbohydrate) (δC/δH 50−95/2.4−5.8) regions in the 2D HSQC NMR spectrum of AL.

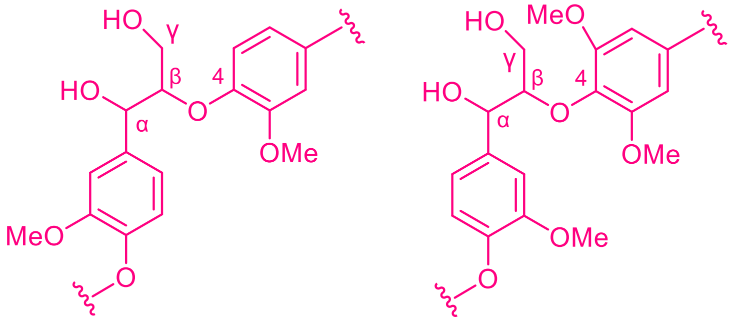

Aα
β-O-4
β Aryl ether in G and S units

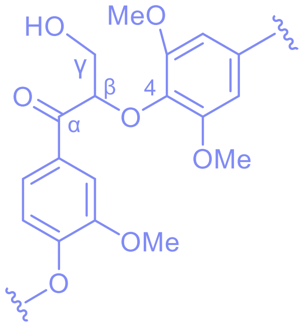

A(α-keto)
β-O-4
β Aryl ether in S units (keto)

R-α
β-β'
Resinol

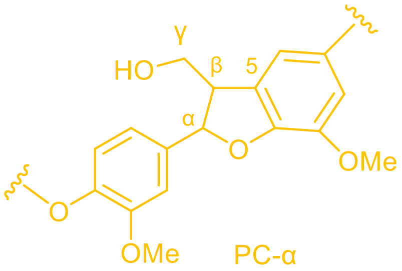

PC-α
β-5
Phenylcoumaran

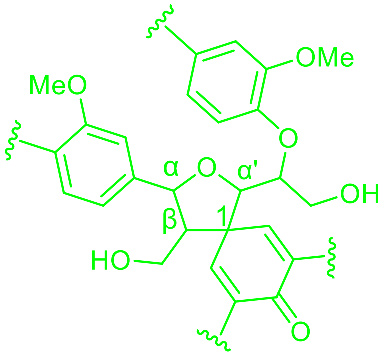

SP-α
β-1
Spirodienone

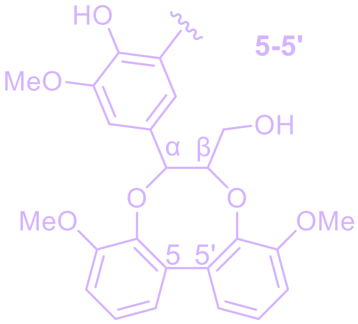

D-α
5-5'
Dibenzodioxacin

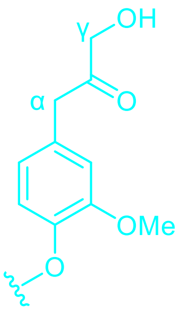

HK
(Hibbert's ketone)

Figure S4. Main structural units identified in AL.

Table S2. Signal assignments of side chain structures in AL.[17,43]

Interunit linkages	Description	δC	δH	%
β-O-4 (α-OH)	β Aryl ether in G and S units	75.2-69.7	5.1-4.6	63.0
	β Aryl ether in G and H units	85.2-82.5	4.5-4.2	
β-O-4 (α-keto)	β Aryl ether in S units with ketone in α	84.5-82.2	5.3-5.0	4.4
**Total β-O-4**				**67.4**
5-5' (D-α)	5-5' in dibenzodioxacin	86.6-83.4	5.0-4.8	6.1
β-β' (R-α)	β-β' in resinol	86.5-84.4	4.8-4.5	6.1
β-1 (SP-α)	β-1 in spirodienone	83.2-81.5	5.0-4.9	2.7
β-5 (PC-α)	β-5 in phenylcoumaran	89.4-86.4	5.6-5.3	13.6
HK	Hibbert's ketone	68.5-66.3	4.2-4.1	4.1
**Total except β-O-4**				**32.6**

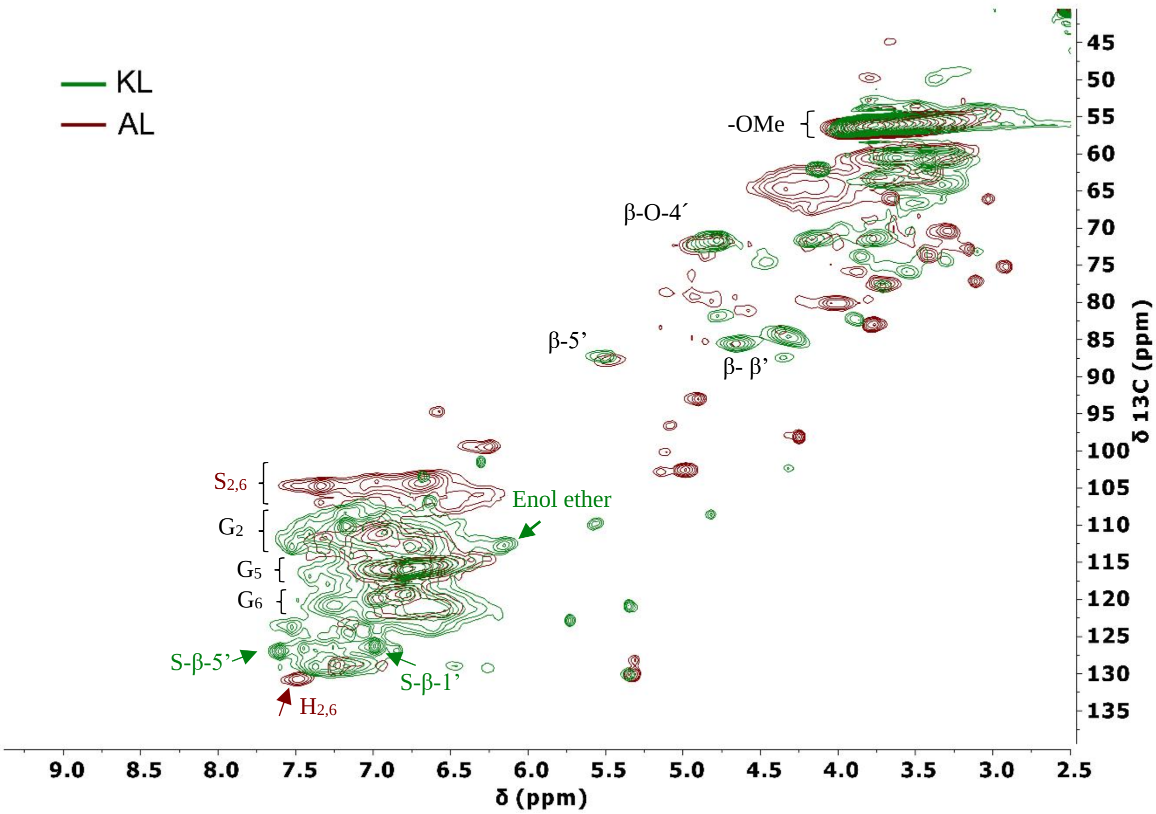

Figure S5. 2D HSQC overlapped spectra of AL (red) and KL (green). The red abbreviations are structures attributed only to AL, the green texts are structures attributed only to KL, the black abbreviations are shared structures present in both lignins.

In the HSQC comparative analysis above, significant differences in lignin structures were observed. Specifically, the AL sample showed signals corresponding to *syringyl* (S2,6) and *p-hydroxyphenyl* (H2,6) units, while the KL sample exhibited almost exclusively *guaiacyl* (G2, G5, G6) (~100%). Additionally, the presence of *stilbene* structures (S-β-5' and S-β-1') was detected in KL. The occurrence of side chains related to stilbene (S-β-5' and S-β-1'), enol-ether, β-5', and β-β' reduces the relative amount of β-O-4' linkages. Significant differences were also found in the carbohydrate region.[54]

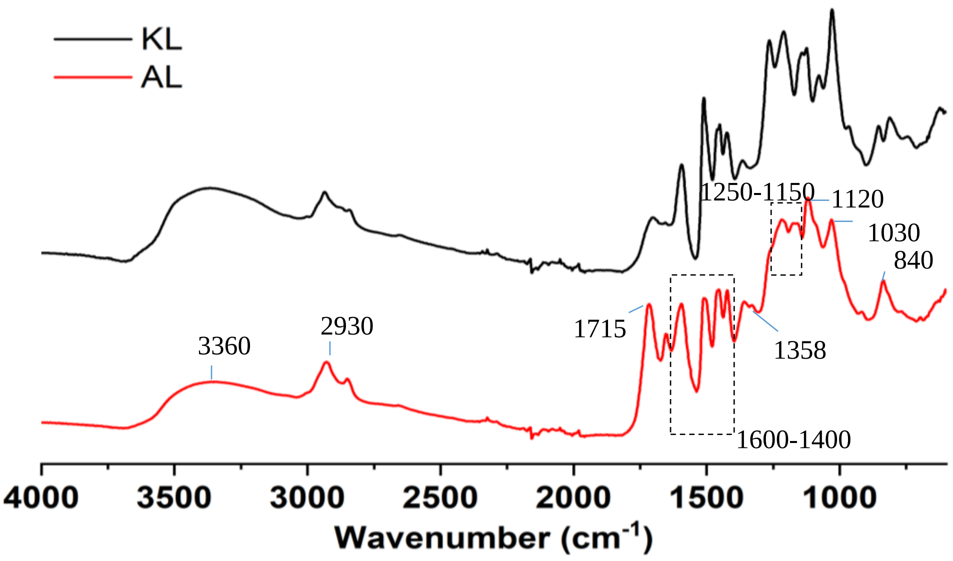

Figure S6. FTIR between KL and AL.

Table S3. Assignments of FT-IR absorption bands for AL.[54]

**Absorption bands (cm^{-1})**	**Assignment**
3360	O-H stretching
2930	Aliphatic C-H stretching
1715	Carbonyl
1600-1400	Aromatic skeletal vibration
1358	Syringyl ring with C-O stretching
1250-1150	C-O stretching
1120	Aromatic C-H in-plane deformation for syringyl type
1030	Aromatic C-H in-plane deformation for guaiacyl type
840	Aromatic C-H bending vibration

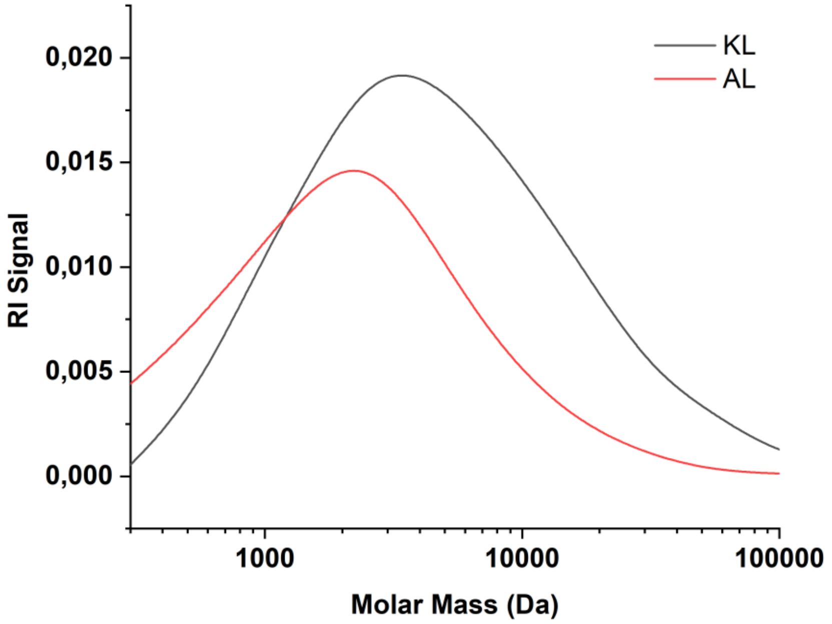

Figure S7. SEC between KL and AL.

Table S4. Molecular weights (Mn and Mw) and polydispersity index (Đ) of AL and KL calculated with SEC.

	**AL**	**KL**
M_n (g/mol)	1460 ± 370	1580 ± 250
M_w (g/mol)	4650 ± 1400	8500 ± 250
Đ	3.1 ± 0.3	6.1 ± 0.5

2.2. Allylation procedure 1 (Zr catalysis + allyl alcohol)

AL (100 mg) was reacted together with allylic alcohol (400 uL, 342 mg) in presence of $Zr(Cp)_2(CF_3SO_3)_2$·THF (zirconocene triflate) (24 mg, 8 mol%) in 2-Me-THF (600 uL). The reaction was carried out at 40 °C. After 6 h the solvents were removed by using a rotary evaporator and the residual catalyst was extracted with DCM (3 x 1 mL). The resulting dark brown solids were dried and analyzed by NMR and FT-IR. The scaled-up procedure was performed with the following quantities: 1.1 g of AL, 4 mL allyl alcohol, 100 mg zirconocene triflate catalyst, 6 mL 2-Me-THF.[42]

2.3. Allylation procedure 2

Allylated lignin was synthesized following a standard procedure.[8] AcetoSolv-L (100 mg) was mixed with a solution of ethanol and sodium hydroxide (5.5 mL, 60:40 volume ratio) in a round-bottomed flask equipped with a condenser. The mixture was stirred at 65°C until a homogeneous dark-brown solution formed. Allyl chloride (0.2 g, 210 µL) was then added dropwise, and the reaction stirred vigorously for 40 hours. After cooling, 5 mL of deionized water was added, causing the precipitated allylated lignin to form. The product was filtered, washed with deionized water, dissolved in acetone (3 x 2 mL), precipitated in deionized water (20 mL), and freeze-dried to obtain a light-brown fine powder (≈ 900 mg). The final product was finally dried in vacuum oven at 50°C for 20 hours completed the process.

2.4 Allylation procedure 3

AL (100 mg) was placed in separate 10 mL pressure vials. Tetrabutylammonium bromide (TBAB) catalyst and diallyl carbonate (DAC) were added (1 and 3 eq. per $mmol_{-OH}/g_{lignin}$ respectively). The pressure vials were sealed and magnetically stirred at 120°C for 5 h. The reaction mixture was then cooled to room temperature, dissolved in ethyl acetate (EtOAc) (5 x 3 mL), and subjected to liquid-liquid extraction with water to recover TBAB. Following extraction, the organic phase was concentrated, precipitated in cyclohexane, and the resulting precipitate (≈ 800 mg) was filtered, washed, and dried under vacuum at 50°C for 24 hours.[40,41]

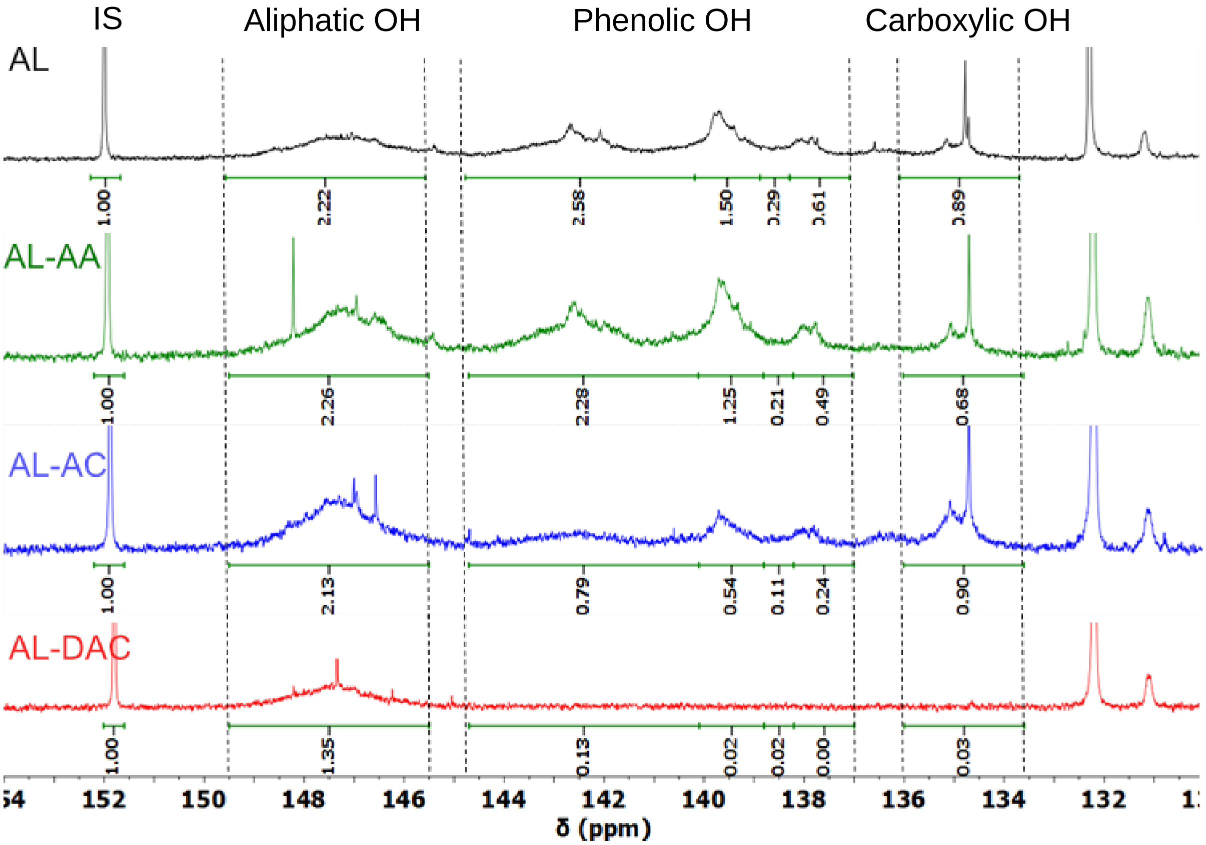

Figure S8. ^{31}P NMR of AL samples before and after allylation procedures: AL unmodified (black), AL-AA (green), AL-AC (blue), AL-DAC (red). N-hydroxy-5-norbornene-2,3-dicarboximide was used as IS (see section 1.2.1 of ESI).

Table S5. Detailed quantification of OH groups and allyl groups before and after allylation calculated with ^{31}P NMR analysis.

	Aliphatic	Condensed phenolic	Non-condensed phenolic	Catechols	4-hydroxy-phenyl	Carboxylic acids	Total –OH	Total Allyl	Yield
	mmol/g	mmol/g	mmol/g	mmol/g	mmol/g	mmol/g	mmol/g	mmol/g	$\frac{mmol_{allyl}}{mmol_{OH}}$
**AL**	1.27	1.47	0.85	0.17	0.35	0.51	4.61	-	
**AL-AA**	1.29	1.31	0.62	0.10	0.40	0.39	4.09	0.52	0.12
**AL-AC**	1.21	0.45	0.31	0.06	0.14	0.51	2.68	1.93	0.58
**AL-DAC**	0.77	0.07	0.01	0.01	0.00	0.02	0.88	3.73	0.81

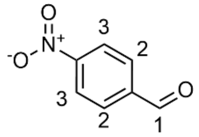

4-nitrobenzaldehyde (IS)

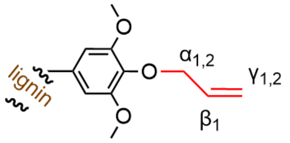

Allylated AL

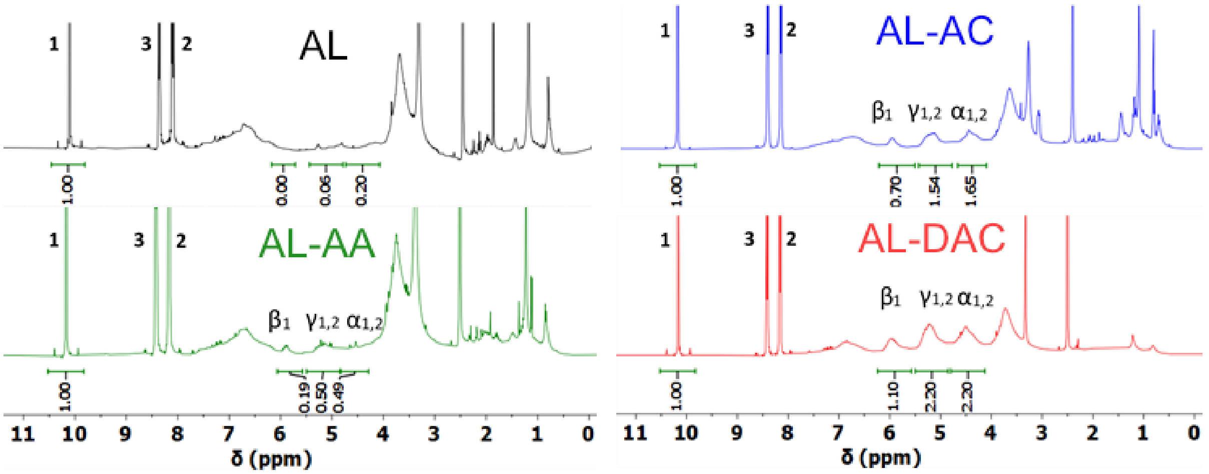

Figure S9. 1H NMR of AL samples before and after allylation procedures. Unmodified AL (black), AL-AA (green), AL-AC (blue), AL-DAC (red). 4-Nitrobenzaldehyde was used as IS (see section 1.2.1 of ESI).

Allyl groups from ^{1}H NMR were calculated as follows:

$$Allyl\ group\ \left(\frac{mmol}{g_{lignin}}\right) = \frac{Internal\ Standard\ \left(\frac{mmol}{g_{lignin}}\right) * Integral\ Allyl\ H_{peak}}{Integral\ Internal\ Standard\ H_{peak}}$$

Table S6. Detailed quantification of allyl groups ^{31}P NMR analysis.

	(mmol/g_{lignin})	Integral allyl peak β_1	Integral internal standard	(mmol/g_{lignin})
**AL-AA**	2.78	0.19	1	0.53
**AL-AC**	2.78	0.70	1	1.95
**AL-DAC**	2.78	1.10	1	3.06

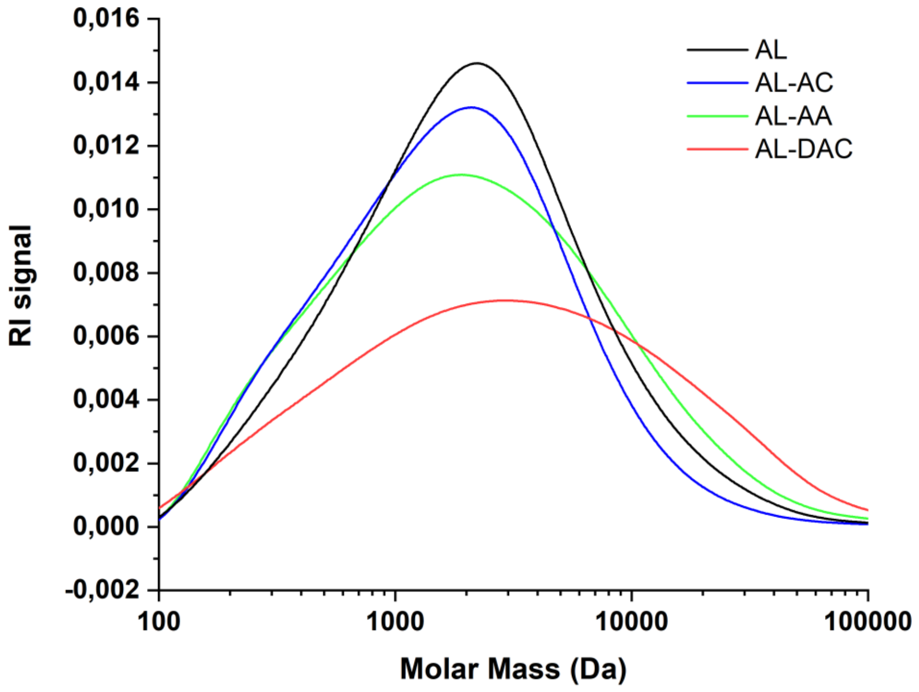

Figure S10. SEC comparison of the allylated lignin samples.

Table S7. Molecular weights (Mn and Mw) and polydispersity index (Đ) of AL before and after allylation procedures calculated with SEC.

	**AL**	**AL-AA**	**AL-AC**	**AL-DAC**
Mn (g/mol)	1460 ± 374	1430 ± 70	1400 ± 65	2020 ± 33
Mw (g/mol)	4650 ± 1500	5660 ± 800	4560 ± 330	11550 ± 1100
D	3.13 ± 0.33	3.95 ± 0.45	3.28 ± 0.09	5.76 ± 0.38

3. Thermosets

3.1 Thermoset formulations and characterization

Trimethylolpropane tris(3-mercaptopropionate), a tri-thiol cross-linker, was used to create thermosets with allylated lignin. The thiol and ene functional groups were kept at a 1:1 molar ratio. To prepare the thermoset resins, around 100 mg of allylated acetosolv lignin (≈60 wt.%) was mixed with 60 mg of the tri-thiol cross-linker (≈40 wt.%). For thorough mixing, 150 mg of butyl acetate was added as a cosolvent. The mixture was then poured into soft silicon molds to facilitate easy removal of the final brittle samples. The resins were left in the fume hood for 24 hours, then moved to an oven and cured at 125 °C for 20 hours. After curing, the thermosets were cooled to room temperature and carefully removed from the molds.

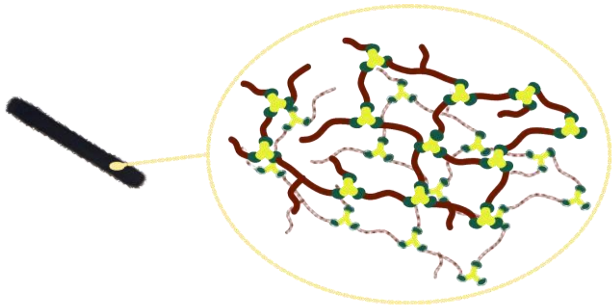

Figure S11. Representation of the final thermoset.

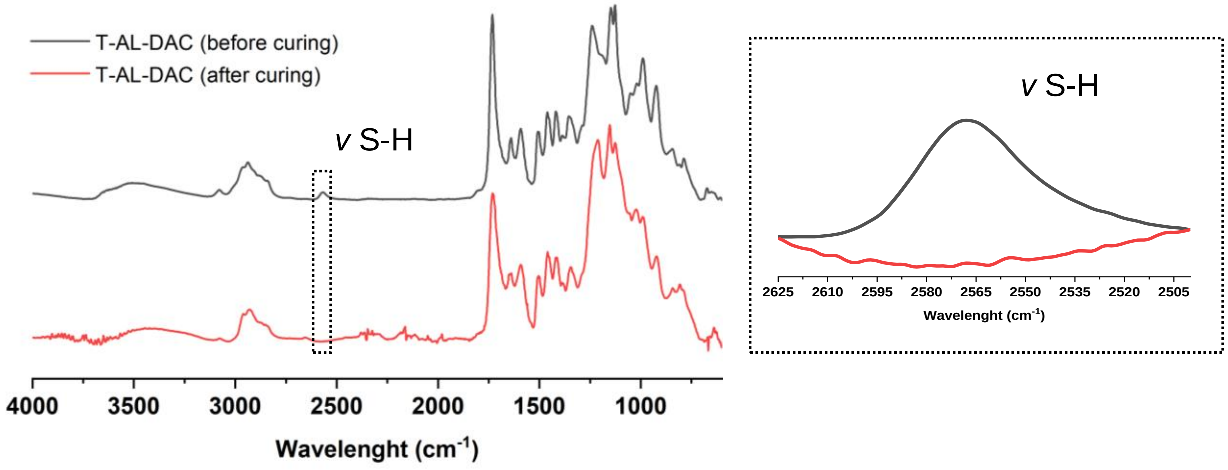

Figure S12. FTIR of AL-DAC mixed with 3TMP crosslinker after solvent evaporation (T-AL-DAC before curing), and T-AL-DAC after 20 hours of thermal curing (T-AL-DAC after curing).

3.2. WAXS characterization

WAXS data, performed on thermoset T-DAC-AL, were fit using Gaussians functions using Origin Software. The width (w) was fixed as parameter, while peak position (xc) and area (A) were calculated by using fitting function.

Table S8. Gaussian models and parameters of WAXS data for T-DAC-AL.[41,51]

**Model**	Gaussian			
**Equation**	y = y0 + A/(w*sqrt(pi/(4*ln(2)))) * exp(-4*ln(2)*(x-xc)^2/w^2)			
**Interaction**	Lignin superstructure D1	T-shape D2 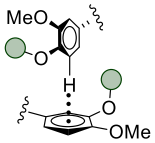	Sandwich D3 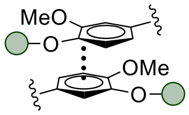	Thioether D4
**xc (peak) (nm^{-1})**	5.29 ± 0.09	9.73 ± 0.22	14,58 ± 0.02	20,57 ± 0.13
**xc (peak) (Å)**	11.88 ± 0.21	6.45 ± 0.15	4.3 ± 0.005	3.05 ± 0.02
**A (area) (nm^{-1})**	30.82 ± 2.78	81.18 ± 12.70	2105,48 ± 24.24	203,82 ± 9.71
**w (width) (nm^{-1})**	1.91	4.0	5,96	5,10
**R = A_S/A_T**	26:1			
**ΔR**	±4			
**Reduced Chi-Sqr**	78.88			
**R-Square (COD)**	0.99			
**Adj. R-Square**	0.99			

The conversion from reciprocal xc (nm^{-1}) to their values in real space, xc (Å), was calculated by using the following equation:

$$xc\ (\text{Å}) = \frac{2\pi}{xc\ (nm^{-1})} * 10$$

The sandwich/T-shape ratio (R) was calculated by calculation the fraction between the 2 areas of their respective Gaussian (A_S and A_T). The error of this ratio (ΔR) was calculated by using the following equation:

$$\Delta R = R * \sqrt{\left(\frac{\Delta A_S}{A_S}\right)^2 + \left(\frac{\Delta A_T}{A_T}\right)^2}$$

ΔA_S and ΔA_T corresponds to A_S and A_T errors, respectively.

4. References

53. D.S Argyropoulos, N. Pajer, and C. Crestini, *J. Vis. Exp*, **2021**, *174*, e62696.

54. Pereira, A., et al., *Biomacromolecules*, **2017**, *18*, 1322-1332
